# *FNTB* Promoter Polymorphisms Are Independent Predictors of Survival in Patients with Triple Negative Breast Cancer

**DOI:** 10.3390/cancers14030468

**Published:** 2022-01-18

**Authors:** Hagen Sjard Bachmann, Dominik Jung, Theresa Link, Anna Arnold, Eva Kantelhardt, Christoph Thomssen, Pauline Wimberger, Martina Vetter, Jan Dominik Kuhlmann

**Affiliations:** 1 Institute of Pharmacology and Toxicology, Centre for Biomedical Education and Research (ZBAF), Witten/Herdecke University, 58453 Witten, Germany; hagen.bachmann@uni-wh.de (H.S.B.); dominik.jung@uni-wh.de (D.J.); anna.arnold@uni-wh.de (A.A.); 2 Institute of Pharmacogenetics, University Hospital Essen, University of Duisburg-Essen, 45122 Essen, Germany; 3 Department of Gynecology and Obstetrics, Medical Faculty, University Hospital Carl Gustav Carus, Technische Universität Dresden, 01307 Dresden, Germany; theresa.link@uniklinikum-dresden.de (T.L.); pauline.wimberger@uniklinikum-dresden.de (P.W.); 4 National Center for Tumor Diseases (NCT), German Cancer Research Center (DKFZ), Faculty of Medicine, University Hospital Carl Gustav Carus, Technische Universität Dresden, Helmholtz-Zentrum Dresden-Rossendorf (HZDR), 01307 Dresden, Germany; 5 German Cancer Consortium (DKTK), Partner Site Dresden and German Cancer Research Center (DKFZ), 69120 Heidelberg, Germany; 6 Department of Gynecology, Martin Luther University Halle Wittenberg, 06120 Halle, Germany; eva.kantelhardt@uk-halle.de (E.K.); christoph.thomssen@uk-halle.de (C.T.); martina.vetter@uk-halle.de (M.V.); 7 Institute of Medical Epidemiology, Bioinformatics and Statistics, Martin Luther University Halle-Wittenberg, 06120 Halle, Germany

**Keywords:** breast cancer, *FNTB*, rs3215788, rs11623866, rs192403314, TNBC, single nucleotide polymorphism

## Abstract

**Simple Summary:**

In breast cancer, the promising efficacy of farnesyltransferase inhibitors (FTIs) in preclinical studies is in contrast to only limited effects in clinical trials. Therefore, this study focussed on the clinical relevance of polymorphisms in *FNTB*, the gene encoding the catalytically active β-subunit of farnesyltransferase, in early breast cancer. This is the first study on breast cancer suggesting that *FNTB* promoter polymorphisms are independent prognostic biomarkers, particularly in patients with early triple negative breast cancer (TNBC), and possibly modulate *FNTB* transcriptional activity. Taken together, we describe for the first time, a link between *FNTB* promoter polymorphism and the prognosis of breast cancer patients. We propose that *FNTB* genotyping, which is easily possible from a single blood drawing, may allow independent prognostic stratification, particularly in TNBC, in order to identify patients with a high risk of recurrence and poor prognosis. Ultimately, our results encourage further prospective evaluations of the role of *FNTB* promoter polymorphisms in predicting response to FTIs, particularly in TNBC patients.

**Abstract:**

In breast cancer, the promising efficacy of farnesyltransferase inhibitors (FTIs) in preclinical studies is in contrast to only limited effects in clinical Phase II–III trials. The objective of this study was to explore the clinical relevance of farnesyltransferase β-subunit (*FNTB*) single nucleotide promoter polymorphisms (*FNTB*-173 6G > 5G (rs3215788), -609 G > C (rs11623866) and -179 T > A (rs192403314)) in early breast cancer. *FNTB* genotyping was performed by pyrosequencing in 797 patients from a prospective multicentre observational PiA trial (NCT 01592825). In the total cohort, the *FNTB*-173 6G > 5G polymorphism was an independent predictor of RFI (HR = 0.568; 95% CI = 0.339–0.949, *p* = 0.031), OS (HR = 0.629; 95% CI = 0.403–0.980, *p* = 0.040) and BCSS (HR = 0.433; 95% CI = 0.213–0.882; *p* = 0.021), whereas the *FNTB*-609 G > C polymorphism was an independent predictor of RFI (HR = 0.453; 95% CI = 0.226–0.910, *p* = 0.026) and BCSS (HR = 0.227; 95% CI = 0.075–0.687, *p* = 0.009). Subtype analysis revealed the independent prognostic relevance of *FNTB* promoter polymorphisms, particularly in TNBC but not in luminal or HER2-positive intrinsic subtypes. Finally, we used electrophoretic mobility shift assays (EMSAs) to confirm in vitro that the polymorphism *FNTB*-173 6G > 5G resulted in the differential binding of nuclear proteins from five different breast cancer cell lines. This is the first study on breast cancer suggesting that *FNTB* promoter polymorphisms (i) are independent prognostic biomarkers, particularly in patients with early TNBC, and (ii) could modulate *FNTB*’s transcriptional activity.

## 1. Introduction

Breast cancer is one of the major causes of cancer-related deaths worldwide. Standard treatment approaches for breast cancer are dependent on the underlying subtype and can comprise surgery, radiation, chemotherapy, endocrine therapy and targeted therapy, such as the HER-2 directed antibody trastuzumab. However, despite recent advances in early detection and systemic treatment, about 20–30% of patients with early breast cancer experience distant metastatic relapse, which constitutes the predominant cause of breast cancer-specific death [1,2,3,4]. Therefore, the identification of innovative predictive and prognostic biomarkers for personalized treatment of breast cancer is of high clinical interest.

RAS oncogenes encode membrane-associated small guanosine triphosphate (GTP)ases that transduce signals from activated membrane receptors to downstream kinases [5]. For exerting their signalling activity, RAS proteins need post-translational modification, such as farnesylation, which confers them with lipophilic properties and allows their attachment to the cell membrane [6]. Therefore, an important and rate-limiting step in RAS activation is farnesylation by the RAS-modifying enzyme farnesyltransferase. Farnesyltransferase, together with geranylgeranyltransferase-I, belongs to the class of CAAX-prenyltransferases, which mediate prenylation at the C-terminal cysteine residues of target proteins. Both CAAX-prenyltransferases share a common α-subunit but have their own β-subunits. The β-subunit of farnesyltransferase has a size of 46 kDa and mediates the transfer of a farnesyl group from farnesyl diphosphate to its target proteins [7].

**Figure 1:**
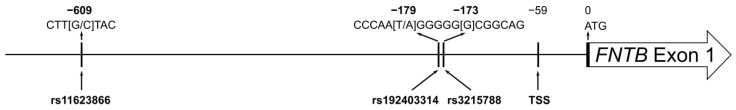
The FNTB promotor region. The schematic representation shows the local sequences and positions relative to ATG. The polymorphisms of interest are indicated.

The RAS signalling axis is often (over)activated in cancer, and RAS mutations are frequently observed in tumours [8]. Human breast cancer principally has a very low frequency of RAS mutations. However, frequent aberrations in the upstream or downstream elements of the RAS pathway strongly suggest the high relevance of this pathway in breast cancer, suggesting a potential therapeutic role for farnesyl transferase inhibitors (FTIs) in breast cancer patients [9,10]. However, the promising efficacy of FTIs in preclinical approaches [11,12,13] is in contrast to the only limited or absent benefits of FTI treatment in breast cancer patients in clinical trials, in which FTIs were combined with endocrine therapy or chemotherapy [14,15,16,17]. It was shown, for instance, that the FTI tipifarnib in combination with letrozole does not improve the objective response rate or survival compared with letrozole alone in oestrogen receptor (ER)-positive advanced breast cancer [14]. Although different translational approaches to FTI response prediction have been described, such as a two-gene classifier (RASGRP1/APTX) in patients with acute myeloid leukaemia (AML), there is still no reliable biomarker available for predicting the FTI response [18]. All these observations clearly point to a gap in our understanding of the complex function of the farnesyltransferase β-subunit and its associated signalling network. In a previous study, we have already discovered that the genetic variability of the *FNTB* gene locus, encoding the catalytically active β-subunit of farnesyltransferase, is linked to transcriptional regulation of the farnesyltransferase β-subunit and could predict the response to the FTI lonafarnib in ovarian cancer patients in the multi-centre AGO-OVAR-15 Phase II trial [19]. In this regard, we have determined the size and localization of the *FNTB* promoter core region and analysed all the genetic variants within 1000 bp 5′ upstream of the translation start point. Three of these variants (*FNTB*-173 6G > 5G (rs3215788); *FNTB*-609 G > C (rs11623866); *FNTB*-179 T > A (rs192403314)) achieved a minor allele frequency of at least 1 (Figure 1). Finally, we have shown that the C allele of the *FNTB*-609 G > C polymorphism is associated with reduced *FNTB* promoter activity and *FNTB* mRNA expression compared with the G allele. Moreover, the adverse effects of lonafarnib were restricted to patients carrying the homozygous G/G genotype [19]. 

However, the functional and clinical relevance of *FNTB*’s genetic variability in the context of breast cancer is completely unknown. Therefore, the objective of this study was to explore the clinical relevance of the single nucleotide promoter polymorphisms of *FNTB* (*FNTB*-173 6G > 5G; *FNTB*-609 G > C; *FNTB*-179 T > A) in a comprehensive breast cancer cohort (*n* = 797) and to investigate how the genetic variability of the *FNTB* promoter may affect the binding of transcription factors.

## 2. Materials and Methods

### 2.1. Patient Characteristics

Fresh frozen primary tumour specimens (*n* = 797) from women with a histologically confirmed primary diagnosis of invasive carcinoma of the breast (International Classification of Disease-Oncology [ICD-O-3] codes C50.0–9) without evidence of distant metastasis were collected as part of the multicentre prospective PiA cohort (NCT 01592825) at the Martin Luther University Halle Wittenberg between 2009 and 2011. The study was approved by the ethics committee of the Martin Luther University Halle Wittenberg (15 September 2009 and 10 March 2010) for patient recruitment (15 September 2016 for this subprotocol) and informed consent was obtained from each patient. Tumour specimens were frozen fresh after primary surgery and stored at −80 °C until further use. Clinical and pathological parameters were obtained for each patient and documented using SPSS 24 (SPSS Inc., Chicago, IL, USA). The TNM-staging system was used [20]. Patient information was pseudo-anonymized prior to analysis. Receptor-defined breast cancer subtypes were determined according to the St. Gallen classification [21]. Due to missing Ki-67 values, we used histopathological grading to assess cell proliferation [22].

### 2.2. Polymorphism Retrieval and Bioinformatic Analyses

Analysis of the putative rs3215788 and rs11623866 allele-dependent binding sites of transcription factors was performed with MatInspector [23] and Alibaba2.1 [24]. Predictions were based on comparisons between matrices representing different human transcription factors and the sequences of the major or minor allele, yielding a matrix similarity value. Positive and negative deviations from the optimized matrix similarity indicated greater and smaller likelihoods of an actual in vivo transcription factor binding, respectively.

### 2.3. DNA Preparation and Determination of FNTB Genotypes

The genomic DNA of primary breast cancer tissue was purified with the QIAamp DNA Mini Kit (Qiagen, Hilden, Germany). For polymerase chain reaction (PCR), the Taq DNA Polymerase Master Mix RED (Ampliqon, Herlev, Denmark) was used. Genotypes of the rs11623866, rs192403314 and rs3215788 polymorphisms were determined by restriction fragment length polymorphism analyses using the “slowdown” PCR [25]. The PCRs were performed with the following primers: rs11623866 (*FNTB*-609 G > C) forward: 5′-GCGGACTGACTGTCTATTT-3′, reverse: 5′-GACGCCGTCTCAGTATCA -3′, resulting in a PCR product of 140 bp; rs192403314 (*FNTB*-179 T > A) and rs3215788 (*FNTB*-173 6G > 5G) forward: 5′-GCAGCAGCTCCTCTGCCCAA-3′, reverse: 5′- ACTCGAGCGGGCTAAAGC-3′, resulting in a PCR product of 137 bp. Amplified fragments harbouring the rs11623866 locus were digested with the restriction enzyme RsaI (New England Biolabs, Beverly, MA, USA) by incubation for 90 min at 37 °C. RsaI specifically cuts PCR products that carry the G allele (101 + 39 bp). Fragments harbouring the rs3215788 locus were analogously digested with the restriction enzyme BslI (New England Biolabs, Beverly, MA, USA) by incubation for 90 min at 55 °C. BslI specifically cuts PCR products that carry the 6G allele (111 + 26 bp). Adequate negative and positive controls were routinely used to ascertain correct genotyping. The accuracy of genotyping was validated by direct sequencing of 20 randomly selected samples. Additionally, pyrosequencing was used for validation of the rs11623866 and rs3215788 genotypes and to determine the genotype regarding rs192403314 on a PyroMark Q24 pyrosequencer according to the manufacturer’s instructions. Briefly, amplification of the samples, using the same PCR primers sequences as before, with the reverse primers containing a 5′-biotin modification, yielded a 140 bp and a 137 bp biotinylated PCR product, respectively. The biotinylated PCR product was captured on streptavidin-coated sepharose beads, and the forward and backward strands were separated using a PyroMark Q24 Vacuum Workstation. Sequencing of the biotinylated single-strand DNA was performed using the following sequencing primers: 5′-TTCACTTATCCTTGTTCCT-3′ (rs11623866) and 5′-CAGCTCCTCTGCCCAA-3′ (rs3215788 and rs192403314). All pyrosequencing results revealed 100% concordance with the previous results obtained by PCR product digestion. Since blood leucocytes for single nucleotide polymorphism analysis were not available from most of the patients, we performed genotyping in the DNA from primary breast cancer tissue in our study. In 42 of our patients, we additionally genotyped DNA from matching blood leucocytes and obtained concordant genotypes (tumour DNA vs. blood leucocyte DNA) in >95% of cases. This confirmed that our genotyping results represent constitutive changes in the “germline” and not somatic alterations in the tumour.

### 2.4. Electrophoretic Mobility Shift Assay

Nuclear extracts of the breast cancer cell lines MCF-7, T-47D, BT-474, BT-20 and MDA-MB-231 were prepared using the NuCLEAR Extraction Kit (Sigma, Deisenhofen, Germany), according to the manufacturer’s instructions. The sequences of unlabelled and DY-682 fluorescence-labelled oligonucleotides (Eurofins MWG Operon, Ebersberg, Germany) of the double-stranded DNA probes used were as follows:

5′-TCCTCTGCCCAAT(G)GGGGGCGGCAGCATCTCA-3′

5′-TGAGATGCTGCCGCCCCC(C)ATTGGGCAGAGGA-3′

EMSAs were carried out with the Odyssey^®^ Infrared EMSA Kit (Li-COR Bioscience, Lincoln, NE, USA) according to the manufacturer’s instructions. Double-stranded DNA probes and nuclear extracts of the respective cell line (9–11 µg) were incubated with 2 µL of 10× binding buffer, 2 µL of a mixture of 2.5 mM DTT and 2.5% Tween-20, 1 µg of poly (dI–dC) and 1 µL of 200 mM EDTA (pH 8) in a total volume of 20 µL for 30 min at room temperature and separated by electrophoresis on a 4% polyacrylamide gel in 1× Tris–Borate-EDTA running buffer. The gels were scanned by direct infrared fluorescence detection on the Odyssey imaging system (Li-COR Bioscience, Lincoln, NE, USA).

### 2.5. Statistical Analysis

All statistical analyses were performed using GraphPad Prism 8.3 (GraphPad Software, La Jolla, CA, USA) and SPSS software version 25 (IBM, Armonk, NY, USA). The standardized definitions for efficacy end points (STEEP) criteria were used as endpoint definitions [26]. The primary endpoints of this study were recurrence-free interval (RFI), breast cancer-specific survival (BCSS) and overall survival (OS). Women without an event were right-censored at the date of last contact. Information on survival was obtained in 2021. Clinical variables and genotypes were compared using Pearson’s Chi^2^ test for categorical data. As a post-hoc test for parameters showing a significant association in the omnibus Pearson’s Chi^2^ test, we used a comparison of the column proportions adjusted by Bonferroni correction. Kaplan–Meier plots and the log-rank test for trend were used to retrospectively evaluate the relationship between the rs3215788 (6G > 5G) and rs11623866 (G > C) genotypes and the outcome between the date of primary diagnosis and the end of follow-up. Both univariate analysis and stepwise backward multivariable Cox regression analysis were used to analyse the effect of the genotypes of the *FNTB* promoter polymorphisms on clinical outcomes. For multivariate analyses, the Cox model was adjusted for clinical and pathological parameters with the classification into subgroups given in Supplementary Tables S1–S3. Hazard ratios (HR) and 95% confidence intervals (95% CI) were calculated on the basis of the Cox regression model. Differences with *p*-values < 0.05 were considered significant; all *p*-values were two-tailed.

## 3. Results

### 3.1. Association of FNTB Promoter Polymorphisms with Clinical and Pathological Parameters of Breast Cancer

*FNTB* genotyping was performed by pyrosequencing in 797 patients from a representative cohort of non-metastasized early breast cancer patients (51.7% pT1, 42.9% pT2, 4.6% pT3, 0.8% pT4; 76% luminal, 13.9% HER2-positive, 10% triple negative breast cancer, TNBC), recruited in the prospective multicentre observational PiA trial (NCT 01592825).

With regard to the FNTB-173 6G > 5G promoter polymorphism, 80/797 patients (10%) showed a homozygous 5G/5G genotype, whereas 261/797 patients (32.7%) were heterozygous (6G/5G) and 456/797 patients (57.2%) exhibited a homozygous 6G/6G genotype, which is in line with the global genotype frequencies [27]. Subsequently, we compiled the FNTB-173 6G > 5G genotypes with the patients’ clinical and pathological data. We observed a correlation between FNTB-173 6G > 5G and the histopathological grading at borderline significance (*p* = 0.048). Post-hoc testing indicated that, within the G1 subgroup, the proportion of heterozygous patients (6/5) was greater than the proportion of patients homozygous for the major allele (6/6). However, this correlation was not considered significant after Bonferroni correction (adjusted *p*-value = 0.053). There was no correlation with other clinical and pathological parameters, such as tumour stage, intrinsic breast cancer subtype or histology. Interestingly, we observed a numerical trend that patients with a homozygous 5/5 genotype were more likely to be PR-negative. However, this trend did not reach statistical significance in our patient cohort (*p* = 0.081; Supplementary Table S1). 

Concerning the FNTB-609 G > C promoter polymorphism, 156/797 patients (19.6%) had a homozygous C/C genotype, whereas 336/797 patients (42.2%) were heterozygous (G/C) and 305/797 patients (38.3%) showed a homozygous G/G genotype, which is consistent with the global genotype frequencies [27]. We observed the association of the FNTB-609 G > C genotype with tumour size (*p* = 0.036) and grading (*p* = 0.031). Interestingly, there was a statistically significant association between FNTB-609 G > C and PR status (*p* = 0.009). Post-hoc testing revealed that the proportion of PR-negative patients with a C/C genotype was greater than the proportion of PR-negative patients with a G/G genotype (adjusted *p*-value = 0.007). Conversely, the proportion of PR-positive G/G genotype carriers was greater than the proportion of PR-positive patients with a C/C genotype (adjusted *p*-value = 0.007). There was no correlation with other clinical or pathological parameters (Supplementary Table S2).

Concerning the FNTB 179 T > A promoter polymorphism, a homozygous AA genotype was non-detectable in our patient cohort; a heterozygous T/A variant was present in only 13/797 patients (1.6%), whereas the majority of patients (784/797, 98.4%) exhibited a homozygous T/T genotype. This was comparable with the global genotype distribution [27]. Despite an association with tumour stage (*p* = 0.016), there was no correlation of the FNTB-179 T > A polymorphism with other clinical and pathological parameters (Supplementary Table S3).

Conclusively, we report, for the first time, a link between FNTB promoter polymorphisms, particularly FNTB-609 G > C, and PR status in breast cancer patients. Further analysis of the FNTB-179 T > A promoter polymorphism was discontinued in this study, due to the extreme rarity of the A allele and the absence of a homozygous A/A genotype in our study cohort.

### 3.2. Univariate Prognostic Relevance of FNTB Promoter Polymorphisms and Their Association with Triple Negative Breast Cancer

According to univariate Cox regression analyses in the total patient cohort with RFI, OS or BCSS as separate outcome variables, the FNTB-173 6G > 5G polymorphism predicted OS (HR = 0.641, 95% CI = 0.412–0.997, *p* = 0.048) and BCSS (HR = 0.486, 95% CI = 0.240–0.981, *p* = 0.044) at borderline statistical significance (Table 1), whereas FNTB-609 G > C was prognostically non-informative (Table 2).

Since we observed the association of FNTB promoter polymorphisms with PR status (refer to Section 3.1), we re-performed the univariate Cox regression analysis after stratifying the patients according to the intrinsic subtypes of breast cancer. Interestingly, the univariate prognostic relevance of FNTB-173 6G > 5G was evident for RFI (HR = 0.214, 95% CI = 0.049–0.925, *p* = 0.039) and OS (HR = 0.201, 95% CI = 0.047–0.865, *p* = 0.031) in TNBC but not in any other subtype, suggesting that the univariate prognostic relevance of FNTB-173 6G > 5G in the total cohort can predominantly be ascribed to the contribution of the TNBC subgroup (Table 1). In all cases, the homozygous FNTB-173 6G/6G genotype was prognostically inferior compared with the FNTB-173 6G/5G genotype.

For the FNTB-609 G > C polymorphism, which was prognostically non-informative for RFI, OS and BCSS in the total patient cohort, its prognostic relevance with regard to OS became evident in TNBC (HR = 0.197, 95% CI = 0.043–0.900, *p* = 0.036) but not in any other subtype (Table 2). In this analysis, patients with the homozygous FNTB-609 G/G genotype had worse outcome compared with those with a homozygous FNTB-609 C/C genotype.

**Figure 2:**
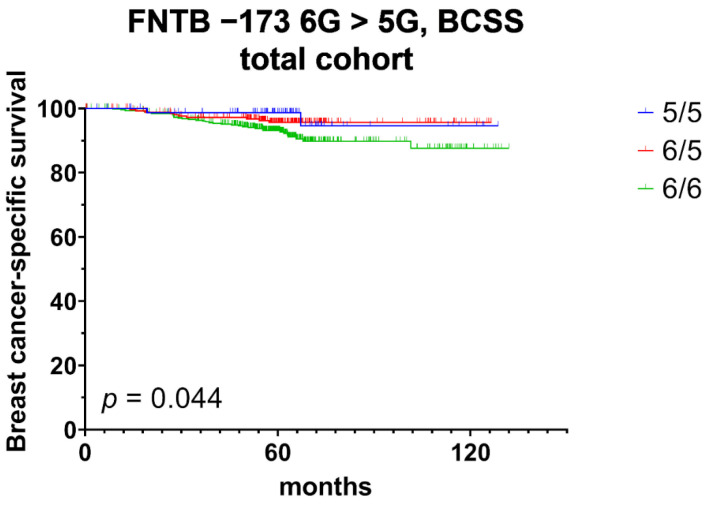
Kaplan–Meier analysis of breast cancer-specific survival (BCSS) in the total patient cohort stratified by promoter polymorphism rs3215788 (FNTB-173 6G > 5G) genotypes.

**Figure 3:**
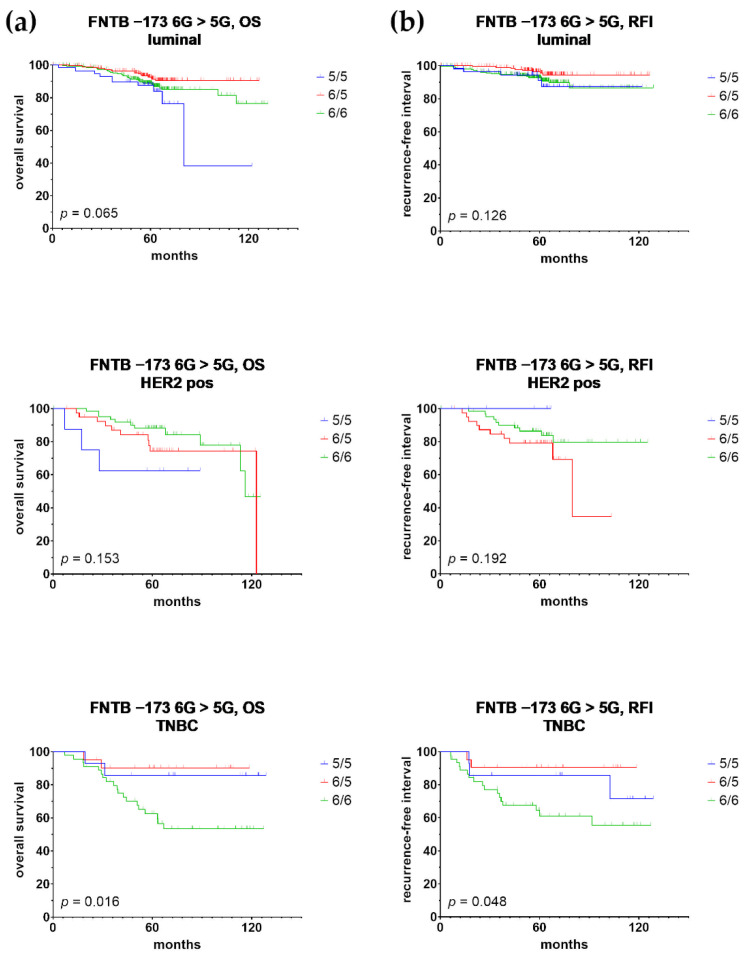
Kaplan–Meier analysis of overall survival (OS, (**a**)) and recurrence-free interval (RFI, (**b**)) in patients with luminal, HER2-positive and triple-negative breast cancer, stratified by promoter polymorphism rs3215788 (FNTB-173 6G > 5G) genotypes.

We additionally performed Kaplan–Meier analysis. While FNTB-173 6G > 5G predicted only BCSS at borderline statistical significance in the total patient cohort (*p* = 0.044; Figure 2), this polymorphism was a prognostic factor for RFI (*p* = 0.048) and OS (*p* = 0.016), exclusively in the TNBC cohort (Figure 3). A homozygous 6/6 genotype clearly exhibited the worst survival, both in the total cohort and in the TNBC subgroup. The FNTB-609 G > C polymorphism did not show any prognostic relevance in the Kaplan–Meier analysis, neither in the total cohort nor among subtypes.

In summary, we demonstrated the univariate prognostic relevance of the promoter polymorphisms FNTB-173 6G > 5G and FNTB-609 G > C, which became particularly evident in patients with TNBC.

### 3.3. Multivariate Prognostic Relevance of FNTB Promoter Polymorphisms and Their Association with Triple Negative Breast Cancer

According to the multivariate Cox regression analysis in the total cohort, adjusted for established breast cancer risk factors (i.e., age, tumour size, histopathological grading, histology, ER- PR- and HER2 receptor status and breast cancer subtype; see Supplementary Tables S1–S3) we observed that the *FNTB*-173 6G > 5G polymorphism was an independent predictor of all the outcome parameters investigated (RFI: HR = 0.568, 95% CI = 0.339–0.949, *p* = 0.031; OS: HR = 0.629, 95% CI = 0.403–0.980, *p* = 0.040; BCSS: HR = 0.433, 95% CI = 0.231–0.882, *p* = 0.021; Table 1). Moreover, the *FNTB*-609 G > C polymorphism was an independent prognostic factor of RFI (HR = 0.453, 95% CI = 0.226–0.910; *p* = 0.026) and BCSS (HR = 0.227, 95% CI = 0.075–0.687; *p* = 0.009; Table 2).

According to subtype analysis, we observed the independent prognostic relevance of both promoter polymorphisms in TNBC only, with *FNTB*-173 6G > 5G as an independent predictor of RFI (HR = 0.214, 95% CI = 0.049–0.925, *p* = 0.039) and OS (HR = 0.201, 95% CI = 0.047–0.865, *p* = 0.031; Table 1), whereas the *FNTB*-609 G > C polymorphism was an independent predictor of OS (HR = 0.197, 95% CI = 0.043–0.900; *p* = 0.036; Table 2). 

To conclude, we report the independent prognostic relevance of the FNTB-609 G > C and FNTB-173 6G > 5G promoter polymorphisms in the total patient cohort and particularly in TNBC patients.

### 3.4. The FNTB-173 6G > 5G Polymorphism Confers Genotype-Specific Binding of Breast Cancer Cell Line-Derived Nuclear Protein to the FNTB Promoter Region

**Figure 4:**
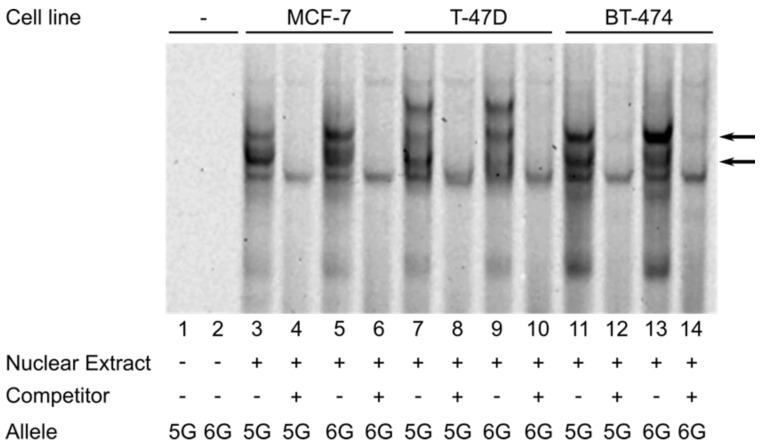
Electromobility shift assay (EMSA) revealing allele-dependent binding of nuclear proteins from different breast cancer cell lines to the locus of rs3215788 (*FNTB*-173 6G > 5G). Bands resulting from specific DNA–protein binding disappeared upon incubation with an excess of unlabelled competitor oligonucleotides. Consistently for all cell lines examined, one specific band (upper arrow) was clearly stronger with the major 6G allele than with the 5G allele; conversely, another specific band (lower arrow) was more pronounced after incubation with the 5G allele.

We evaluated whether the *FNTB*-173 6G > 5G polymorphism has an influence on the binding of nuclear proteins, which, in turn, could modulate *FNTB*’s transcriptional activity. Therefore, we performed an electrophoretic mobility shift assay (EMSA) using nuclear extracts of three luminal breast cancer cell lines (MCF-7, T-47D and BT-474, Figure 4). While we did not detect nuclear protein bands binding exclusively to only one allele, we observed two bands with differing intensities for the 6G and the 5G allele. This suggested that at least two different nuclear proteins differentially bind to the 5G or 6G allele, respectively. We subsequently confirmed these results in two different TNBC cell lines (BT-20, MDA-MB-231; Supplementary Figure S1).

Next, we aimed to identify the proteins potentially involved in these distinct binding patterns. Therefore, we performed in silico transcription factor binding prediction using MatInspector in order to identify the transcription factors predicted to bind preferentially to the 6G or 5G allele. The first search using the default settings did not predict considerable differences in transcription factor binding to the 6G and 5G alleles. Therefore, we repeated the search using reduced matrix similarity values (opt.—0.05), as was justified by our differential in vitro results and obtained a list of differentially binding transcription factors (Table 3), with GLIS3 being ranked as the most likely candidate to preferentially bind the 6G allele and with ZNF658 to preferentially bind the 5G allele.

## 4. Discussion

The germline genetic variability of the *FNTB* locus and its clinical relevance for breast cancer is still an open question, particularly since promising preclinical data on FTIs are in contrast to the mostly unsuccessful clinical trials [11,12,13,14,15,16,17]. We, for first time, analysed clinical relevance of *FNTB* promoter polymorphisms in breast cancer patients and demonstrated the independent prognostic relevance of *FNTB*-173 6G > 5G and *FNTB*-609 G/C. This result was derived from a retrospective analysis of a comprehensive cohort of 797 patients with early, i.e., non-metastasized, breast cancer from the multicentre prospective PiA cohort (NCT 01592825), in which we have previously defined the clinical relevance of caspase 8 polymorphisms and their association with tumour infiltrating lymphocytes [28]. From the clinical translational point of view, our data strongly suggest that *FNTB* promoter polymorphisms could be useful for independent prognostic stratification in primary non-metastasized breast cancer patients in order to identify patients with a high risk of recurrence and poor prognosis. Farnesylation of RAS by farnesyltransferase is a rate-limiting step in the activation of the RAS signalling pathway [6]. Although breast cancer usually exhibits a very low frequency of RAS mutations, aberrations in the upstream or downstream elements of this pathway are common, which suggests the RAS pathway as a potential therapeutic target for breast cancer patients [9,10]. The functionality of the *FNTB*-609 G/C promoter has already been proposed by us in a previous study on ovarian cancer, in which we experimentally demonstrated, by reporter assays, that the -609 G allele is associated with increased *FNTB* transcription [19]. In line with these findings, breast cancer patients with a homozygous -609 G/G genotype in the present study had worse RFI or BCSS compared with patients with a homozygous -609 C/C genotype. Therefore, we suggest that the -609 C/C genotype promotes *FNTB* transcriptional activity, RAS farnesylation and RAS signalling, which, in turn, may contribute to a more aggressive breast cancer phenotype with a high risk of relapse and poor prognosis. 

Considering that patients with a *FNTB*-173 6G/6G genotype exhibit an inferior prognosis compared with heterozygous 6G/5G patients for all the investigated outcome parameters (RFI, OS, BCSS), a similar conceptual framework can be assumed for the *FNTB*-173 6G > 5G promoter polymorphism. Here again, we suppose that, in particular, the homozygous *FNTB*-173 6G/6G genotype is associated with increased farnesyltransferase activity, a more active RAS signalling pathway and an aggressive breast cancer phenotype with poor outcome. Functional reporter assays for *FNTB* -173 6G/5G were beyond the scope of this explorative study. Nevertheless, we experimentally demonstrated herein that this polymorphism affects the binding spectrum of the nuclear proteome to the *FNTB* promoter region in luminal and triple negative breast cancer cell lines and is therefore likely to modulate *FNTB* transcriptional activity, comparable with *FNTB*-609 G/C. Our in silico analysis corroborated this hypothesis and predicted that a subfamily member of the Krüppel-like zinc finger transcription factors, named GLIS3, preferentially binds the *FNTB*-173 6G allele but not the 5G allele. GLIS transcription factors are regulators of a number of physiological processes, such as pancreatic β-cell development [29] and differentiation of human embryonic stem cells [30]. Interestingly, overexpression of GLIS3 has already been reported in breast cancer tissue [31]. Furthermore, we predicted the transcription factor ZNF658 to preferentially bind the *FNTB*-173 5G allele. ZNF658 belongs to the Cys2His2 class of zinc finger proteins, which accounts for about 3% of the human genome and is the largest class of putative transcription factors. The majority of these zinc finger proteins have unknown or diverse properties, pointing to a vast regulatory network of these transcription factors that is largely unstudied [32]. Since patients carrying a 5G allele, to which ZNF658 preferentially binds, were prognostically favourable, it is possible that ZNF658 may act as an inhibitory transcription factor that diminishes *FNTB* transcription. However, further in vitro experiments, which were beyond the scope of the present translational study, will be needed in order to confirm the putative role of GLIS3 and ZNF658 in *FNTB*’s transcriptional regulation and its effects on oncogenic RAS signalling in breast cancer.

TNBC is a particularly aggressive form of breast cancer and accounts for 10–15% of all breast cancer cases. Due to absent ER, PR and HER2/neu expression, there is ongoing effort towards a molecular subtyping of TNBC for risk stratification [33,34]. Most interestingly, the subtype analysis in our study revealed that prognostic capacity of the investigated *FNTB* promoter polymorphisms could selectively be ascribed to TNBC patients and was not detectable in hormone receptor-positive breast cancer patients. This suggests that *FNTB*’s genetic variability could be highly informative in terms of risk stratification in TNBC patients and might complement our current knowledge on the intrinsic TNBC subtypes and their associated risk profiles [34]. Furthermore, *FNTB* may exert further unknown tumour-promoting functions in TNBC that relate to the biology of tumour stem cells. In this regard, it was shown in vitro that low-dose tipifarnib in triple negative MDA-MB-231 cells inhibit neither cell growth nor the activity of the RAS pathway but it suppressed a HIF-driven aggressive stem cell phenotype in these cells [35].

The absent prognostic relevance of *FNTB* polymorphisms in hormone receptor-positive breast cancer patients could possibly be explained by the fact that RAS signalling, for which the activity of farnesyltransferase is rate-limiting, has a rather subordinate role in this type of breast cancer. Nevertheless, since the mechanistic background of farnesyltransferase signalling was beyond the scope of our present study, further in vitro experiments will be warranted in order to decode *FNTB*-associated signalling networks and their contribution to the malignant progression of breast cancer.

## 5. Conclusions

Taking all our findings together, we describe, for the first time, a link between *FNTB* promoter polymorphisms and breast cancer prognoses. We propose that *FNTB* genotyping, which is easily possible from a single blood drawing, may allow independent prognostic stratification, particularly in TNBC patients, in order to identify patients with a high risk of recurrence and poor prognosis. Ultimately, our results encourage further prospective evaluations of the role of *FNTB* promoter polymorphisms in predicting the response to FTIs, especially in TNBC patients.

## Figures and Tables

**Table 1:** Prognostic impact of the FTNB-173 promoter polymorphism rs3215788 (6G > 5G) for genotype 6/5 vs. 6/6 in univariate und multivariate analyses of RFI, OS and BCSS.

**Endpoint**	**Subgroup**	**Univariate Cox Regression**				**Multivariate Cox Regression**			
	(Patients; Events)	*p*-Value	HR	95% CI		*p*-Value	HR	95% CI	
*RFI*	total cohort (*n* = 797; 83)	0.056	0.607	0.364	1.013	**0.031**	**0.568**	**0.339**	**0.949**
	luminal (*n* = 606; 41)	0.056	0.465	0.212	1.021	-	-	-	-
	HER2 positive (*n* = 111; 20)	0.170	1.857	0.768	4.494	*0.854*	*1.094*	*0.420*	*2.853*
	TNBC (*n* = 80; 17)	**0.039**	**0.214**	**0.049**	**0.925**	**0.039**	**0.214**	**0.049**	**0.925**
*OS*	total cohort (*n* = 797; 114)	**0.048**	**0.641**	**0.412**	**0.997**	**0.040**	**0.629**	**0.403**	**0.980**
	luminal (*n* = 606; 67)	0.085	0.596	0.330	1.074	-	-	-	-
	HER2 positive (*n* = 111; 24)	0.288	1.594	0.674	3.770	*0.958*	*0.976*	*0.390*	*2.442*
	TNBC (*n* = 80; 23)	**0.031**	**0.201**	**0.047**	**0.865**	**0.031**	**0.201**	**0.047**	**0.865**
*BCSS*	total cohort (*n* = 797; 47)	**0.044**	**0.486**	**0.240**	**0.981**	**0.021**	**0.433**	**0.213**	**0.882**
	luminal (*n* = 606; 19)	0.078	0.328	0.095	1.134	-	-	-	-
	HER2 positive (*n* = 111; 11)	0.543	1.447	0.440	4.751	*0.643*	*0.722*	*0.182*	*2.868*
	TNBC (*n* = 80; 17)	0.089	0.276	0.063	1.217	0.089	0.276	0.063	1.217

Abbreviations: HR: hazard ratio; CI: confidence interval; RFI: recurrence-free interval; OS: overall survival; BCSS: breast cancer-specific survival; TNBC: triple negative breast cancer. Significant results (*p* < 0.05) highlighted in bold. In cases where the coefficients did not converge, the values are shown in italics.

**Table 2:** Prognostic impact of the FTNB-609 promoter polymorphism rs11623866 (G > C) for genotype C/C vs. G/G in univariate und multivariate analyses of RFI, OS, and BCSS.

Endpoint	*Subgroup*	Univariate Cox Regression				Multivariate Cox Regression			
	(Patients; Events)	*p*-Value	HR	95% CI		*p*-Value	HR	95% CI	
*RFI*	total cohort (*n* = 797; 83)	0.196	0.637	0.322	1.261	**0.026**	**0.453**	**0.226**	**0.910**
	luminal (*n* = 606; 41)	0.355	0.648	0.259	1.625	-	-	-	-
	HER2 positive (*n* = 111; 20)	0.965	0.964	0.186	4.980	*0.171*	*0.265*	*0.039*	*1.777*
	TNBC (*n* = 80; 17)	0.099	0.333	0.090	1.230	0.099	0.333	0.090	1.230
*OS*	total cohort (*n* = 797; 114)	0.645	0.883	0.520	1.498	-	-	-	-
	luminal (*n* = 606; 67)	0.826	0.942	0.555	1.600	-	-	-	-
	HER2 positive (*n* = 111; 24)	0.156	2.219	0.738	6.670	*0.694*	*0.769*	*0.207*	*2.853*
	TNBC (*n* = 80; 23)	**0.036**	**0.197**	**0.043**	**0.900**	**0.036**	**0.197**	**0.043**	**0.900**
*BCSS*	total cohort (*n* = 797; 47)	0.082	0.386	0.132	1.129	**0.009**	**0.227**	**0.075**	**0.687**
	luminal (*n* = 606; 19)	0.278	0.431	0.094	1.973	-	-	-	-
	HER2 positive (*n* = 111; 11)	0.814	0.761	0.079	7.329	*0.104*	*0.077*	*0.003*	*1.702*
	TNBC (*n* = 80; 17)	0.070	0.144	0.018	1.172	0.070	0.144	0.018	1.172

Abbreviations: HR: hazard ratio; CI: confidence interval; RFI: recurrence-free interval; OS: overall survival; BCSS: breast cancer-specific survival; TNBC: triple negative breast cancer. Significant results (*p* < 0.05) highlighted in bold. In cases where the coefficients did not converge, the values are shown in italics.

**Table 3:** List of transcription factors predicted to bind preferentially to the 6G or 5G allele of the rs3215788 locus (*FNTB* -173 6G > 5G).

	Transcription Factor	Opt.	Strand	Matrix Similarity	Mat. Sim.—Opt.	Sequence
**Major allele only (6G)**	BSAP/PAX5	0.87	(+)	0.869	−0.001	cctctgcccAATGgggggcggcagcatct
	ZIC1	0.76	(+)	0.742	−0.018	ctgcccaatggGGGGcggc
	GLIS3	0.88	(−)	0.885	0.005	gccgCCCCccattgggc
	KLF15	0.91	(+)	0.876	−0.034	cccaatggGGGGcggcagc
	PATZ1	0.89	(+)	0.850	−0.040	caatggggGGCGgcagcatctcaccagacca
	ZBTB14	0.89	(+)	0.857	−0.033	aatggggGGCGgcagca
**Minor allele only (5G)**	ZNF658	0.75	(−)	0.702	−0.048	cgCCCCcattgggcaga

Red letters within the sequence highlight the highly conserved positions; capital letters denote the core sequence.

## Data Availability

The manuscript contains all the relevant data.

## Supplementary Materials

The following supporting information can be downloaded at: https://www.mdpi.com/article/10.3390/cancers14030468/s1, Figure S1: Electromobility shift assay (EMSA) revealing allele-dependent binding of nuclear proteins from triple negative breast cancer cell lines to the locus of rs3215788 (FNTB -173 6G > 5G; Table S1: Association of the FNTB -173 6G > 5G promoter polymorphism with the patient’s clinical and pathological data; Table S2: Association of the FTNB -609 G > C promoter polymorphism with the patient’s clinical and pathological data; Table S3: Association of the FTNB -179 T > A promotor polymorphism with the patient’s clinical and pathological data.
