# Supplementary material for: FNTB Promoter Polymorphisms Are Independent Predictors of Survival in Patients with Triple Negative Breast Cancer
Source: Cancers (Basel). 2022 Jan 18;14(3):468. doi: 10.3390/cancers14030468 (PMC8833514; doi:10.3390/cancers14030468)
Supplement: Supplementary file 1 [file cancers-14-00468-s001.zip › Supplementary Figure S1.pptx]

## Slide 1
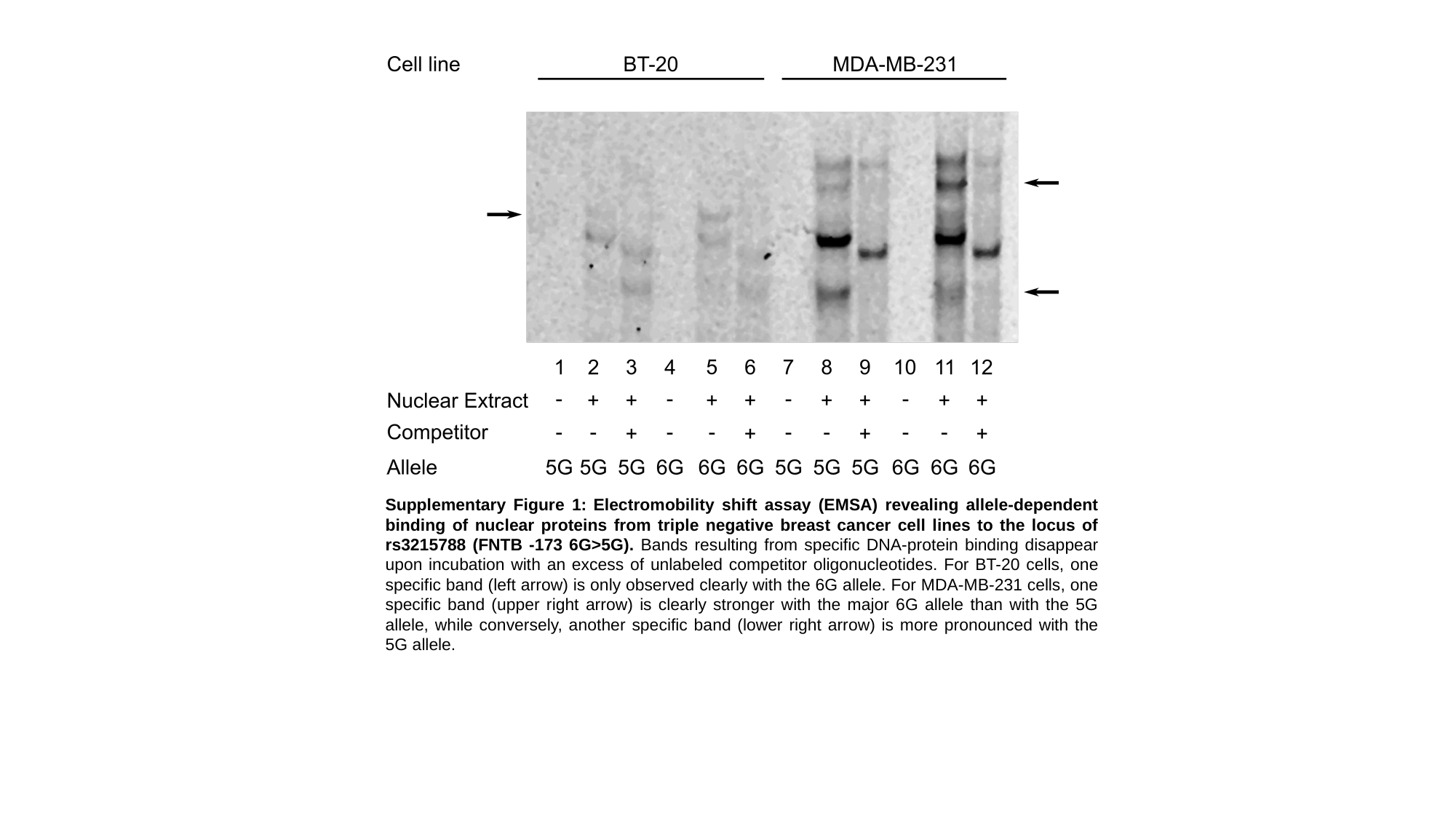

Supplementary Figure 1: Electromobility shift assay (EMSA) revealing allele-dependent binding of nuclear proteins from triple negative breast cancer cell lines to the locus of rs3215788 (FNTB -173 6G>5G). Bands resulting from specific DNA-protein binding disappear upon incubation with an excess of unlabeled competitor oligonucleotides. For BT-20 cells, one specific band (left arrow) is only observed clearly with the 6G allele. For MDA-MB-231 cells, one specific band (upper right arrow) is clearly stronger with the major 6G allele than with the 5G allele, while conversely, another specific band (lower right arrow) is more pronounced with the 5G allele.
