# Supplementary material for: FNTB Promoter Polymorphisms Are Independent Predictors of Survival in Patients with Triple Negative Breast Cancer
Source: Cancers (Basel). 2022 Jan 18;14(3):468. doi: 10.3390/cancers14030468 (PMC8833514; doi:10.3390/cancers14030468)
Supplement: Supplementary file 1 [file cancers-14-00468-s001.zip › Supplementary table S1 with caption and post hoc testing.pdf]

Supplementary Table S1: Association of the *FNTB* -173 6G>5G promoter polymorphism with the patient's clinical and pathological data

										post-hoc comparisons of column proportions[a]		
			5/5		6/5		6/6			5/5	6/5	6/6
	**Number**	**%**	**n**	**%**	**n**	**%**	**n**	**%**	***p*-value***	**(A)**	**(B)**	**(C)**
**Total**	797	100.0	80	10.0	261	32.7	456	57.2				
**Age**												
< 35 years	15	1.9	0	.0	6	2.3	9	2.0				
35 - 50 years	178	22.3	21	26.3	58	22.2	99	21.7				
> 50 years	604	75.8	59	73.8	197	75.5	348	76.3	.652			
**Mean Age (Min - Max)**	62.0	(22 - 90)	63.0	(35 - 87)	62.0	(25 - 90)	62.0	(22 - 89)				
**Tumorsize**												
< 2 cm	412	51.7	43	53.8	137	52.5	232	50.9				
2 - 5 cm	354	44.4	32	40.0	116	44.4	206	45.2				
>5 cm	31	3.9	5	6.3	8	3.1	18	3.9	.704			
**Tumor stage**												
pT1	412	51.7	43	53.8	137	52.5	232	50.9				
pT2	342	42.9	31	38.8	110	42.1	201	44.1				
pT3	37	4.6	5	6.3	12	4.6	20	4.4				
pT4	6	.8	1	1.3	2	.8	3	.7	.960			
**Grading**												
G1	91	11.4	8	10.0	40	15.3	43	9.4		n.s	n.s	n.s
G2	496	62.2	44	55.0	154	59.0	298	65.4		n.s	n.s	n.s
G3	210	26.3	28	35.0	67	25.7	115	25.2	**.048**	n.s	n.s	n.s
**Histology**												
ductal	639	80.2	66	82.5	199	76.2	374	82.0				
lobular	117	14.7	10	12.5	45	17.2	62	13.6				
other	41	5.1	4	5.0	17	6.5	20	4.4	.405			
**Estrogen receptor status**												
neg.	124	15.6	17	21.3	39	14.9	68	14.9				
pos.	673	84.4	63	78.8	222	85.1	388	85.1	.334			
**Progesteron receptor status**												
neg.	237	29.7	32	40.0	79	30.3	126	27.6				
pos.	560	70.3	48	60.0	182	69.7	330	72.4	.081			
**Hormone receptor status**												
neg.	116	14.6	17	21.3	36	13.8	63	13.8				
pos.	681	85.4	63	78.8	225	86.2	393	86.2	.201			
**HER2 status**												
neg.	686	86.1	72	90.0	221	84.7	393	86.2				
pos.	111	13.9	8	10.0	40	15.3	63	13.8	.482			
**Breast cancer subtype**												
luminal	606	76.0	58	72.5	200	76.6	348	76.3				
HER2 pos.	111	13.9	8	10.0	40	15.3	63	13.8				
TNBC	80	10.0	14	17.5	21	8.0	45	9.9	.142			

*. *p*-values were calculated using the Pearson's Chi^2 test for categorical data. [a] Bonferroni adjustments were used to adjust the *p*-values of all pairwise comparisons. For each significant pair, the key of the smaller category is placed under the category with the larger proportion. n.s. = not significant; significance threshold = 0.05
