# Supplementary material for: FNTB Promoter Polymorphisms Are Independent Predictors of Survival in Patients with Triple Negative Breast Cancer
Source: Cancers (Basel). 2022 Jan 18;14(3):468. doi: 10.3390/cancers14030468 (PMC8833514; doi:10.3390/cancers14030468)
Supplement: Supplementary file 1 [file cancers-14-00468-s001.zip › Supplementary table S2 with caption and post hoc testing.pdf]

Supplementary Table S2: Association of the *FTNB* -609 G>C promoter polymorphism with the patient's clinical and pathological data

										post-hoc comparisons of column proportions[a]		
			CC		GC		GG			CC	GC	GG
	**Number**	**%**	**n**	**%**	**n**	**%**	**n**	**%**	***p*-value***	**(A)**	**(B)**	**(C)**
**Total**	797	100.0	156	19.6	336	42.2	305	38.3				
**Age**												
< 35 years	15	1.9	2	1.3	6	1.8	7	2.3				
35 - 50 years	178	22.3	39	25.0	71	21.1	68	22.3				
> 50 years	604	75.8	115	73.7	259	77.1	230	75.4	.831			
**Mean Age (Min - Max)**	62.0	(22 - 90)	62.0	(28 - 88)	62.0	(25 - 90)	63.0	(22 - 89)				
**Tumorsize**												
< 2 cm	412	51.7	87	55.8	177	52.7	148	48.5		n.s	n.s	n.s
2 - 5 cm	354	44.4	59	37.8	153	45.5	142	46.6		n.s	n.s	n.s
>5 cm	31	3.9	10	6.4	6	1.8	15	4.9	**.036**	**B(.021)**	n.s	n.s
**Tumor stage**												
pT1	412	51.7	87	55.8	177	52.7	148	48.5				
pT2	342	42.9	57	36.5	145	43.2	140	45.9				
pT3	37	4.6	10	6.4	12	3.6	15	4.9				
pT4	6	.8	2	1.3	2	.6	2	.7	.431			
**Grading**												
G1	91	11.4	17	10.9	49	14.6	25	8.2		n.s	**C(.035)**	n.s
G2	496	62.2	88	56.4	205	61.0	203	66.6		n.s	n.s	n.s
G3	210	26.3	51	32.7	82	24.4	77	25.2	**.031**	n.s	n.s	n.s
**Histology**												
ductal	639	80.2	119	76.3	274	81.5	246	80.7				
lobular	117	14.7	26	16.7	47	14.0	44	14.4				
other	41	5.1	11	7.1	15	4.5	15	4.9	.670			
**Estrogen receptor status**												
neg.	124	15.6	29	18.6	58	17.3	37	12.1				
pos.	673	84.4	127	81.4	278	82.7	268	87.9	.102			
**Progesteron receptor status**												
neg.	237	29.7	59	37.8	104	31.0	74	24.3		**C(.007)**	n.s	n.s
pos.	560	70.3	97	62.2	232	69.0	231	75.7	**.009**	n.s	n.s	**A(.007)**
**Hormone receptor status**												
neg.	116	14.6	28	17.9	54	16.1	34	11.1				
pos.	681	85.4	128	82.1	282	83.9	271	88.9	.086			
**HER2 status**												
neg.	686	86.1	136	87.2	286	85.1	264	86.6				
pos.	111	13.9	20	12.8	50	14.9	41	13.4	.789			
**Breast cancer subtype**												
luminal	606	76.0	116	74.4	251	74.7	239	78.4				
HER2 pos.	111	13.9	20	12.8	50	14.9	41	13.4				
TNBC	80	10.0	20	12.8	35	10.4	25	8.2	.551			

*. *p*-values were calculated using the Pearson's Chi² test for categorical data.; [a] Bonferroni adjustments were used to adjust the *p*-values of all pairwise comparisons. For each significant pair, the key of the smaller category is placed under the category with the larger proportion. n.s. = not significant; significance threshold = 0.05
