# Supplementary material for: FNTB Promoter Polymorphisms Are Independent Predictors of Survival in Patients with Triple Negative Breast Cancer
Source: Cancers (Basel). 2022 Jan 18;14(3):468. doi: 10.3390/cancers14030468 (PMC8833514; doi:10.3390/cancers14030468)
Supplement: Supplementary file 1 [file cancers-14-00468-s001.zip › Supplementary table S3 with caption and post hoc testing.pdf]

**Supplementary Table S3:** Association of the *FTNB* -179 T>A promotor polymorphism with the patient's clinical and pathological data

| | | | TA | | TT | | | post-hoc comparisons of column proportions[a] TA | TT |
|---|---|---|---|---|---|---|---|---|---|
| | Number | % | n | % | n | % | *p*-value* | (A) | (B) |
| **Total** | 797 | 100.0 | 13 | 1.6 | 784 | 98.4 | | | |
| **Age** | | | | | | | | | |
| < 35 years | 15 | 1.9 | 0 | .0 | 15 | 1.9 | | | |
| 35 - 50 years | 178 | 22.3 | 1 | 7.7 | 177 | 22.6 | | | |
| > 50 years | 604 | 75.8 | 12 | 92.3 | 592 | 75.5 | .369 | | |
| **Mean Age (Min - Max)** | 62.0 | (22 - 90) | 73.0 | (48 - 87) | 62.0 | (22 - 90) | | | |
| **Tumorsize** | | | | | | | | | |
| < 2 cm | 412 | 51.7 | 5 | 38.5 | 407 | 51.9 | | | |
| 2 - 5 cm | 354 | 44.4 | 6 | 46.2 | 348 | 44.4 | | | |
| >5 cm | 31 | 3.9 | 2 | 15.4 | 29 | 3.7 | .084 | | |
| **Tumor stage** | | | | | | | | | |
| pT1 | 412 | 51.7 | 5 | 38.5 | 407 | 51.9 | | n.s | n.s |
| pT2 | 342 | 42.9 | 5 | 38.5 | 337 | 43.0 | | n.s | n.s |
| pT3 | 37 | 4.6 | 3 | 23.1 | 34 | 4.3 | | **B(.001)** | n.s |
| pT4 | 6 | .8 | 0 | .0 | 6 | .8 | **.016** | n.s | n.s |
| **Grading** | | | | | | | | | |
| G1 | 91 | 11.4 | 4 | 30.8 | 87 | 11.1 | | | |
| G2 | 496 | 62.2 | 6 | 46.2 | 490 | 62.5 | | | |
| G3 | 210 | 26.3 | 3 | 23.1 | 207 | 26.4 | .085 | | |
| **Histology** | | | | | | | | | |
| ductal | 639 | 80.2 | 12 | 92.3 | 627 | 80.0 | | | |
| lobular | 117 | 14.7 | 1 | 7.7 | 116 | 14.8 | | | |
| other | 41 | 5.1 | 0 | .0 | 41 | 5.2 | .506 | | |
| **Estrogen receptor status** | | | | | | | | | |
| neg. | 124 | 15.6 | 1 | 7.7 | 123 | 15.7 | | | |
| pos. | 673 | 84.4 | 12 | 92.3 | 661 | 84.3 | .430 | | |
| **Progesteron receptor status** | | | | | | | | | |
| neg. | 237 | 29.7 | 3 | 23.1 | 234 | 29.8 | | | |
| pos. | 560 | 70.3 | 10 | 76.9 | 550 | 70.2 | .596 | | |
| **Hormone receptor status** | | | | | | | | | |
| neg. | 116 | 14.6 | 1 | 7.7 | 115 | 14.7 | | | |
| pos. | 681 | 85.4 | 12 | 92.3 | 669 | 85.3 | .479 | | |
| **HER2 status** | | | | | | | | | |
| neg. | 686 | 86.1 | 12 | 92.3 | 674 | 86.0 | | | |
| pos. | 111 | 13.9 | 1 | 7.7 | 110 | 14.0 | .513 | | |
| **Breast cancer subtype** | | | | | | | | | |
| luminal | 606 | 76.0 | 11 | 84.6 | 595 | 75.9 | | | |
| HER2 pos. | 111 | 13.9 | 1 | 7.7 | 110 | 14.0 | | | |
| TNBC | 80 | 10.0 | 1 | 7.7 | 79 | 10.1 | .752 | | |

*. *p*-values were calculated using the Pearson's $Chi^2$ test for categorical data. [a] Bonferroni adjustments were used to adjust the *p*-values of all pairwise comparisons. For each significant pair, the key of the smaller category is placed under the category with the larger proportion. n.s. = not significant; significance threshold = 0.05
